# American Association of Obstetricians and Gynecologists

**Published:** 1902-11

**Authors:** 


### SOCIETY PROCEEDINGS.

### American Association of Obstetricians and Gynecologists.

*Fourteenth Annual Meeting, held at Washington, D. C., September 16, 17 and 18, 1902.*

THE President, Dr. Edwin Ricketts of Cincinnati, O., in the chair.

An address of welcome was delivered by Brigadier-General George M. Sternberg, U. S. Army (retired), late Surgeon-General, of Washington. This was responded to by the president.

### DISEASE OF THE PELVIS IN THE YOUNG AND UNMARRIED.

Dr. C. L. Bonifield, of Cincinnati, dealt with gynecological diseases which occurred in women who had never had sexual intercourse. Physicians were now more frequently consulted about such troubles, but this was probably because the modern woman bears discomfort less well than her ancestors. Undeveloped uteri, disorders of menstruation, tuberculous disease of appendages and acute displacements are the most common affections in these patients. When menstruation does not make its appearance at the age it is expected, a thorough investigation of the general condition of the patient is indicated. Pregnancy is always to be borne in mind as a possibility. Dysmenorrhea might be constitutional or local. Local treatment is not required in the former, and the endometritis which causes a majority of the latter can often be relieved by hygienic treatment. Menorrhagia is often caused by anemia, or an interference with the circulation of the liver, heart or kidneys. The uterus in which development has been arrested at the time of puberty is the most important type of undeveloped uterus. It is usually sharply anteflexed, but occasionally retroflexed, and the cervix is long and narrow, and the body dilated. Dysmenorrhea is a prominent symptom. Thorough dilatation, curettage and tight packing, repeated one or more times, if necessary, constitute the best treatment. Tuberculosis of the pelvic peritoneum is often mistaken for typhoid fever or appendicitis. Acute prolapse or retroversion of the uterus may be found as the result of a fall or heavy lifting in young women of lax fiber.

REMOVAL OF THE GALL-BLADDER THROUGH THE LUMBAR INCISION.

Dr. Walter P. Manton, of Detroit, reported the case of a patient 38 years of age, the mother of five children (two abortions), never robust, but able to attend to her domestic duties. She had suffered from a number of gastric attacks, but there had been an absence of symptoms pointing to disease of the biliary tract. Examination showed a loose left kidney, while the right kidney, displaced downward and inward, appeared to be double its normal size and had certain projections which led to the diagnosis of nephroptosis, with probable cystic metamorphosis of the kidney. At the operation, through the nephropexy incision, the fatty capsule of the kidney was found to be embedded in adhesions, which had given rise to the appearance of the enlargement. The kidney which was normal in size and structure, was delivered on the back and placed astride the wound. Below the kidney pouch a distended gall-bladder, containing fluid and nineteen gallstones, the size of hazelnuts, was found surrounded by adhesions. This was enucleated, tied off at the cystic-duct, and removed. The kidney capsule was split and peeled off to the lateral line, fixation sutures were introduced, and the organ was returned to its place. A strip of gauze for drainage was carried from the upper angle of the external wound to the stump of the cystic duct. The patient made a good recovery.

ABDOMINAL AND PELVIC SURGERY AND DRAINAGE.

Dr. Joseph Price, of Philadelphia, said that the more progressive and successful specialists practised most extensive sponge packing or drainage. The modern operator did the same by his gauze pack or the dry operation. A number of operators, doing fairly good work by the suprapubic route, condemned or partially rejected drainage. Some of them, he said, never learned and never would learn how to handle drainage well. After abandoning the suprapubic route, these men were placed in the uncomfortable position of admitting that drainage did what they had refused to do by suprapubic surgery. About all the reported operations were coming from operators opposing drainage or practising it only when they were compelled to do so.

### VAGINAL ROUTE FOR OPERATIONS ON THE UTERUS AND APPENDAGES.

Dr. J. H. Branham, of Baltimore, said that pelvic inflammation was the commonest condition threatening the life of gynecological patients, caused by gonorrhea, abortion, tuberculosis, and other conditions not always discernable. In such the vaginal method of treatment made its best showing. He had operated on more than 100 patients using this method, and had three deaths only. He believes that vaginal drainage is the natural method. It is far more effective and safer. Opening through the vagina was associated with a minimum amount of shock and hemorrhage, and was thus indicated in extreme cases associated with severe toxic symptoms.

### ABDOMINAL SECTION AND THE USE OF ICE.

Dr. F. F. Simpson, of Pittsburg, recommended the local use of ice for the relief of pains to prevent or control peritonitis. He began the use of ice after observing its effects in several hundred cases of pelvic peritonitis of all grades prior to operation. He said:

After having seen many grave cases of this kind yield so favorably to this measure, and having seen a few of them die later from peritonitis following an abdominal section, which removed the bulk of filth, leaving but little behind in a fairly clean cavity, it seemed to me that with the same infecting organisms active in large numbers before operation, and in small numbers after operation, we might expect like results if the same treatment were employed in the two classes of cases. And, further, that by beginning the use of ice before peritonitis had actually developed, we might succeed in preventing that condition.

As to theoretical objections, he said that they disappeared when physicians use ice. No injury to the skin has ever followed its use; the healing of wounds had not been retarded by it, and no depression of the general system had been observed. He ascribed the good effect of cold to its deep penetration. This is based on the known effects of cold on inflammation; on the observations of laboratory workers who had shown that cold actually penetrated deeply into the human cavities and tissues; and upon his own experiments on dogs, which showed that by the local use of ice there could be obtained within the adominal cavity such a degree of cold as would retard the growth of pathogenic bacteria and contract the blood lymph channels, thus depleting the hyperemic areas and checking serous weeping into the peritoneal cavity without damage to the tissues.

### TETANUS FOLLOWING ABDOMINAL SECTION, DUE TO INFECTED LIGATURES. THE ANGIOTRIBE IN ABDOMINAL SURGERY.

Dr. Walter B. Dorsett, of St. Louis, detailed at length two cases which were of women who had undergone ventrofixation of the uterus, or adhesions of the uterus to the surrounding tissues, due to previous inflammatory conditions. The material used for fastening the uterus was kangaroo tendon. This was the source of infection. He had used the angiotribe successfully 25 times, as follows: abdominal hysterectomy, 10 times; hemorrhoids, 1; pus tubes, 5 times; extrauterine pregnancy, 4 times; dermoid cyst, 1; ovariotomy, 3 times, and 1 in a case of vaginal hysterectomy. He concluded that (1) patients upon whom it had been tried suffered less post-operative pain; (2) no adhesions to stumps had followed; (3) no secondary hemorrhage had followed; (4) it can be applied, when two instruments were used, alternately by the operator and the assistant without the fear of the slipping of a ligature knot, and in less time.

### EXPLORATORY LAPARATOMY.

Dr. Walter B. Chase, of Brooklyn, said that physical signs and rational symptoms of certain diseases of the contents of the peritoneal cavity being often insufficient for purposes of diagnosis, surgical intervention might be necessary to ascertain the exact state of affairs. Abdominal section for diagnostic purposes is of comparatively recent origin. Its growth has been progressive, but the limitations as yet are not clearly formulated. In chronic cases there might be ample time for study and analysis, with comparison of the progress and fluctuation of symptoms, which would furnish valuable data for forming deliberate judgment. Surgical intervention was looked upon much more favorably by a discriminating public today than formerly, so that the suggestion by the surgeon that an operation was required found a more ready response by the patient or patient's friends.

RUPTURED PUS TUBES.

Dr. Charles Greene Cumston, of Boston, spoke of two methods of dealing with pus tubes; one by posterior colpotomy, followed by incision and drainage of the sac, while the other was to remove the tube, and if the condition was bilateral to do a total hysterectomy. Drainage of the perforated pyosalpinx through the vagina was naturally the easiest and least dangerous method. The vagina was incised, and then the pyosalpinx and the walls of the pus tube might be united to the vaginal wall by means of forceps. The best treatment for perforated pus tubes is by abdominal incision. The extirpation of perforated pyosalpinx is particularly urgent in those patients in whom drainage by posterior colpotomy had been unsuccessful, and also in tuberculous lesions of the tubes.

PELVIC ABSCESS AND ITS TREATMENT.

Dr. Herman E. Hayd, of Buffalo, spoke of a class of cases in which vaginal incision and drainage, supplemented by curettage, should be first employed to eliminate the pus, and then an abdominal section should be done later to relieve the patient of her suffering. Large collections of pus, low down in the pelvis, in a moribund woman are best evacuated through the vagina. He spoke in reference to strong women who were suffering from acute streptococcic infection, who had high temperatures with great pain and tenderness, and under ordinary circumstances ready to submit to major operations, and in whom an acutely tender mass could be felt low down in the pelvis on one or both sides, at times filling up the cul de sac of Douglas. For these, early vaginal incision was imperative and was without danger. The mass would diminish in size; the danger of rupture would be minimised, the pain and symptoms would subside.

APPRENTICES IN OPERATIVE GYNECOLOGY.

Dr. J. J. G. Williams, of Philadelphia, said that the modern hospital training, so far as he himself had observed, was not sufficient for the practical training in gynecological technic. He spoke of the desirability of the modifications in present hospital arrangements looking towards that end. Radical measures, he thought, were alone justifiable in the treatment of pus tubes and that the abdominal route was to be preferred. The vaginal route was simply a makeshift. Whereas the mortality from this latter procedure might be small, the invalidism was very large. Other institutions and other surgeons usually perfected the work, if possible.

BI-INGUINAL CELIOTOMY FOR RETROVERSIONS.

Dr. A. GOLDSPOHN, of Chicago, in speaking of this operation for complicated retroversions of the uterus, claimed that (1) surgical treatment was advisable, since it could do no harm and its good results would not be modified by gestation; (2) the operations in vogue do not meet the ideal requirements; (3) the evolutionary history of the round ligaments showed them to be the ideal structures to utilise; (4) their best service could be gained through the inguinal canals; (5) the lateral inguinal ring gave ample room for the operative technic, with cutting and without fear of subsequent hernia; (6) the histories of 107 cases showed its efficacy; (7) in all the cases recorded the uterus was retained with pronounced anteversion and good involution following childbirth.

STARVATION TREATMENT FOR APPENDICITIS IRRATIONAL.

Dr. JOHN B. DEAVER, of Philadelphia, said his experience of ninety-eight cases for two and one-half months past had furnished the objections to the rest or starvation treatment. An early operation, preferably in the stage of appendiceal colic, was the only rational procedure he had found, and was the best treatment to reduce the mortality in acute appendicitis. The so-called rest treatment failed to check inflammation of the peritoneal structures and in the majority of cases did harm to the patient. The statistics he presented supported the argument. He was willing to grant that operation in the presence of an acutely inflamed general peritonitis was attended by great risk to life, and therefore it was often wise to defer operation, hoping that the inflammatory process would become localised. This was often his practice; but the starvation plan of treatment promised no more in such cases than the mere common practice of abstaining absolutely from giving opium, keeping the bowels freely open by cathartics, or, as some physicians preferred, a hydragogue cathartic, which was both antiseptic and germicidal, giving nourishment by the rectum, when the stomach was intolerant, and using ice or heat locally in the shape of poultices or hot turpentine stupes.

### PRESIDENTIAL ADDRESS.

The subject was "Our Shortcomings: Let Us Reason Together," delivered by Dr. EDWIN RICKETTS, of Cincinnati. The general practitioner, he said was the backbone of medicine. It should not be otherwise. With just and co-equal considerate relations the best interests of the physician and specialist were not to be severed. With changed relations toward the general practitioner complications were sure to arise. Abdominal surgery and gynecology in the hands of all operators were being accompanied with a higher mortality than was true of that done by special workers. Reasons were advanced why the physician and all those engaged in the practice of specialties should enter the political arena. It would teach them what organisation is, what power is and how it can be wielded for good. It would result in pronounced efforts for the organisation of the medical profession which in due time was to command respectful consideration from the national, state and county governments. Such would make a free and independent profession. The relationship of the neurologist as bearing on special work should be better appreciated. The neurologist should be called in consultation oftener, and he, in turn, should consult with the surgeon before sending any of his patients to an institution for partial or permanent detention. He urged the appointment of a national secretary of medicine, which, he believed, would put medicine on its highest and broadest plane. This would mean dignity, higher regard, influence, and an equitable financial remuneration.

### APPENDICITIS CASES AND THE LESSONS THEY TEACH.

Dr. MILES F. PORTER, of Fort Wayne, Ind., reported eleven cases in detail.

### FOUR CASES ILLUSTRATING THE DIFFICULTIES OF DIAGNOSTICATING APPENDICITIS.

Dr. WILLIAM WOTKYNS SEYMOUR, of Troy, reported these cases in abstract. Case I., had previously been operated upon for appendicular abscess. He found a suppurating solid tumor of the ovary. Temperature at the time of operation was

107°; pulse, 180; recovery. Case II., was a woman with contracted pelvis delivered of a dead child. Twelve days later there were symptoms of inflammation in the right iliac fossa, appendicular or tubal, the result of infection. Operation revealed a suppurating gangrenous fibroid of the right anterior uterine wall, which was enucleated, followed by recovery of the patient. Later he did Cesarean section on this patient; mother and child well seven weeks after operation. In Case III. he was summoned to a case of appendicitis some 14 miles distant in the country. His diagnosis was ovarian cyst with twisted pedicle. The mass had increased twofold since his previous visit to the attendant. Removal of the cyst was followed by recovery of the patient. Case IV., woman, single, age about 22. Pains and intense tenderness in appendicular region began with joint pains. Examination of lungs showed bronchial breathing at left base; the next day diffuse bronchial breathing over both lungs. Appendicular symptoms less marked. Toxemia of some sort; no appendicitis.

### INTRAUTERINE FIBROIDS COMPLICATING PREGNANCY, AND RETAINED PLACENTA ASSOCIATED WITH INTRAUTERINE FIBROIDS COMPLICATING PREGNANCY.

Dr. M. A. Tate, of Cincinnati, collected thirty-nine cases from literature, and reported two personal experiences. Analysing these forty-one cases, he showed that in nine cases the names of reporters were given. In six the tumor became gangrenous; hemorrhage was a prominent symptom, occurring in eighteen cases; three polyps were expelled spontaneously; seven polyps were removed; in three the polyp was not removed; in ten labor was normal; in four labor was difficult; in two the child was destroyed; one was a case of turning, and the other a breech; in four the tumor was discovered before labor, in all of the rest, afterward; four cases were reported in which labor set in before time; two were at the fifth, and two at the seventh month. The following complications were reported: septicemia, eight; measles, one; puerperal mania, one; retained placenta, four cases. Cold applications, iodides, ergot, whiskey, vinegar, packing of uterus with gauze, and removal of tumor. Causes of death: hemorrhage, three; sepsis, three; peritonitis, one; and collapse, one, making in all eight cases. If all of the other cases, including the nine without histories, recovered there would be thirty-three recoveries and eight deaths, a mortality of 19½ per cent.

### ABDOMINAL SECTIONS DURING PREGNANCY.

Dr. J. Henry Carstens of Detroit, had had the following complications of pregnancy: appendicitis, five; hernia, one; fibroids, four; abdominal hysterectomy, one; vaginal hysterectomy, three; ovariotomy, three; and miscellaneous three, or altogether, twenty cases and five deaths; mortality, 25 per cent. This included all his cases for many years back. Today he thought the mortality would be less. Acute diseases which required prompt operation could be operated upon notwithstanding pregnancy. Tumors that would interfere with labor should be operated on. Tumor above the brim of the pelvis, or which could be shoved above the brim of the pelvis, need not be interfered with; still, as a rule, all tumors took on a very rapid growth during pregnancy, and the increase in size might interfere with the various functions of life and then surgical intervention was required.

### DECIDUOMA MALIGNUM.

Dr. Lewis S. McMurtry, of Louisville, reported a case. Clinically, the disease presented a well-defined history. The disease appeared after abortion or labor, the tumor being situated upon the endometrium of the body of the uterus. Of 128 recorded cases, in 40 per cent. the disease appeared after mole pregnancy. Hemorrhage was the first and most conspicuous symptom, and was not controlled by curettage. The discharge was usually offensive, especially in the advanced stage. The disease had a marked tendency to early metastasis; the lungs and vagina were the most common sites for metastatic deposits. The disease was so rapid in its course that the period from first symptoms until the death of the patient was only a few weeks or months. The only successful treatment was the early and complete extirpation of the uterus. In the author's case the disease appeared in a woman of thirty-five immediately after abortion. Persistent hemorrhage and fetid discharge from the uterus prompted operative intervention. The uterus and its appendages were removed by abdominal hysterectomy, and the patient made a prompt recovery.

### PERFORATING ULCER OF THE DUODENUM.

Dr. John B. Murphy, of Chicago, reviewed the etiology, pathology, and diagnosis of duodenal ulcers, and considered the surgical treatment of perforation. An analysis of his twenty collected cases showed: average age in the thirteen cases in which age was stated was thirty-five years. Of the nineteen cases, five were females and fourteen males. Of the twelve cases in which it was stated whether or not there were symptoms present previous to perforation, in nine they were present previous to perforation; in three there were no previous symptoms; in only five cases did the symptoms point to the stomach or duodenum; in six cases it was stated that the perforations were sutured; of these two died, three recovered. In one the result was not stated. In eight cases drainage only was used; of these seven died, and one recovered. He concluded the diagnosis of perforating ulcer was difficult, or, better, practically impossible without an exploratory laparatomy. In many cases there was no evidence of duodenal disease previous to the perforation. The most important physical sign, in addition to those of perforative peritonitis from perforations in other portions of the intestinal tract, was the flatness of the superficial piano percussion note in the right hypochondrium. The leucocytosis in one case, the only one in which it was seen, was pronounced, showed an inflammatory condition, in contradistinction to the usual absence of it in intestinal obstruction and fat necrosis of the pancreas. Leucocytosis, however, was not a necessary manifestation of perforation or of inflammation. It was often entirely absent in typhoid perforations. Collapse was absent in duodenal perforation except when associated with severe hemorrhage. Collapse was always secondary to abrasion or denudation of the endothelial covering of the peritoneum, which abrasion permitted of rapid absorption. In all cases of perforative peritonitis operation should be done at the earliest possible moment after the perforation, and experience showed that the mortality was in direct ratio to the length of time that elapsed between the perforation and the operation. Of thirteen cases operated upon more than thirty hours after perforation, all terminated fatally, while in twelve cases, where less than thirty hours had elapsed, 66 2-3 per cent. recovered. The operation must be complete—that is, it must be pursued to an effective suture of the perforation. Drainage was insufficient, as of eighteen cases treated by drainage alone, all died. The suture of the opening can be easily inserted, as in 98 per cent. of the perforating ulcers into the peritoneum the opening was in the first portion of the duodenum, its most accessible portion. Where duodenal perforation was suspected, the incision should be through the right rectus muscle. It could then be carried upward to the costal arch, or downward to the symphysis pubis without dividing any of the transverse muscles. The incision through the rectus muscle was the one which he commonly made in operating for appendicitis. It could be enlarged upward or downward without interfering with the muscle fibers. Drainage or no drainage was a matter of personal election, influenced, more or less, by the pathological condition present at the time of the operation. The prognosis depended upon the virulence of the peritonitis produced; the time the material had been allowed to remain in the peritoneum; on the presence or absence of blistering or abrasion of the peritoneum at the time of operation.

### PERITONEAL TUBERCULOSIS.

Dr. Rufus B. Hall, of Cincinnati, maintained that tuberculosis of the peritoneum in women is not a rare affection. It occurs often enough to make it necessary to consider it in the differential diagnosis of all obscure diseases in the pelvis and abdomen. In a large majority of cases there were no appreciable manifestations of tuberculosis in other parts of the body. The symptoms simulated several other conditions in the pelvis and abdomen. The diseases most likely to be confounded were the recurrent attacks of appendicitis of the catarrhal form, small fibroid tumors, with old tubo-ovarian disease, and recurrent attacks of pelvic inflammation. If the case is one of tuberculosis, however, the temperature chart will suggest this disease if the record is taken every four hours for a period of ten to fifteen days. In no other condition is there such exact regularity in the afternoon rise of temperature. Cases of tuberculosis of the peritoneum in which there was encysted dropsy, or an accumulation of pus or serum, should be operated on; after the necessary surgical repair the abdomen should be drained. Vaginal drainage in women is preferable, because it gives perfect drainage and prevents ventral hernia.

### SURGICAL TREATMENT OF PERFORATED GASTRIC ULCER WITH GENERAL INFECTION OF THE PERITONEAL CAVITY.

Dr. H. Howitt, of Guelph, Ont., said that acute perforation, with general infection of the abdomen, was usually caused by the acute, round ulcer, but might occasionally take place in the course of a chronic ulcer, especially when it was situated on the anterior wall. All the phenomena of acute perforation might result in either form of ulcer in a more indirect manner by the formation of a localised abscess, which afterward ruptured internally. In peritoneal perforation with general infection medicinal remedies are useless. Early, bold and thorough surgery alone can save the patient. When patient is anesthetised an incision from ensiform cartilage to pubis should at once be made, the bowels eviscerated and protected, then the stomach examined and the perforated part brought out of the wound as far as possible and the field guarded by sponges. The perforation might be excised, but it is generally merely closed with two or more rows of silk sutures. Every pouch and corner in the abdomen should be thoroughly inspected and flushed clean. Drainage tubes are used, not placed in the wound, but through stabs, one at the back in each flank depression below the kidney, and one in the lower abdomen to the right or left of the incision for the pelvis. After the intestines are replaced and omentum is spread over them and fastened below the lower end of the wound with a suture or two, the incision is closed as quickly as possible and dressed. In a desperate condition of the patient, a pint of peptonised milk or other suitable liquid food may be injected into the jejunum during operation.

The author said in conclusion that he was aware that many surgeons strongly objected to evisceration, but he maintained that it was impossible by any other known method to make certain that the cleansing of the peritoneum had been done thoroughly. Imperfect toilet is followed by more shock, and is vastly more dangerous than hours of properly managed evisceration.

### CARCINOMA OF THE CERVIX.

Dr. L. H. Dunning, of Indianapolis, presented a summary of sixty-two cases operated upon by hysterectomy. These cases showed on applying the accepted test of time—five years—that the author had had an encouraging number of cures; they all showed the desirability of early operative procedure. The operative methods employed were indicated, and the author's views of the same were discussed. He reported five cases of carcinoma of the cervix uteri in which the women were alive five years after hysterectomy.

